# *International Journal of Molecular Science* 2019 Best Paper Award

**DOI:** 10.3390/ijms21010046

**Published:** 2019-12-19

**Authors:** International Journal of Molecular Science Editorial Office

**Affiliations:** MDPI, St. Alban-Anlage 66, 4052 Basel, Switzerland; ijms@mdpi.com

The Editors of the *International Journal of Molecular Sciences* have established the Best Paper Award to recognize the most outstanding articles published in the areas of molecular biology, molecular physics, and chemistry that have been published in the *International Journal of Molecular Sciences*. The prizes have been awarded annually since 2012 [1,2,3,4,5,6,7].

We are pleased to announce the “*International Journal of Molecular Science* Best Paper Award” for 2019. Nominations, chosen from all papers published in 2018, were made by the Editorial Board. The awards are issued to reviews and research articles separately. Following a review process by the Editorial Board, three top-voted research articles and the three top-voted reviews as follows, in no particular order, have won “*International Journal of Molecular Science* Best Paper Award” for 2019:

## Research Article Award:

**Vitamin D Receptor Is Necessary for Mitochondrial Function and Cell Health**

Chiara Ricca, Alessia Aillon, Loredana Bergandi, Daniela Alotto, Carlotta Castagnoli and Francesca Silvagno

*Int. J. Mol. Sci.***2018**, *19*(6), 1672; doi:10.3390/ijms19061672

Available online: http://www.mdpi.com/1422-0067/19/6/1672

In addition to the known genomic and non-genomic activity of vitamin D, in the last few years our group has described the mitochondrial effects of the hormone and its receptor VDR, and we highlighted the relevance of the mitochondrial control in proliferation, differentiation, and epithelial-mesenchymal transition.

In this article we investigated the importance of VDR not only in mitochondrial activity and integrity but also in cell health. The silencing of the receptor in different healthy, nontransformed, and cancer cells initially decreased cell growth and modulated the cell cycle. We demonstrated that in silenced cells the increased respiratory activity was associated with the elevated ROS production. In the long run, the absence of the receptor caused the impairment of mitochondrial integrity, and finally, cell death. Our observations reveal that VDR plays a central role in protecting the cell from the excessive respiration and production of ROS that leads to cell damage. Our data were obtained in different human cell types, cancerous and healthy cells; therefore, the novel function discovered by our work can be considered a general feature of vitamin D/VDR role in tissues.

An intriguing consideration about this study is based on the observation that many respiratory chain dysfunctions and deleterious ROS overproduction are recurrent themes in human pathologies, ranging from neurodegenerative diseases to cancer, and may be of paramount importance in aging. The results of this study raise the possibility that the loss of VDR function or a repressed expression of the receptor could be partly responsible or at least could be an adverse event in such diseases.

**Figure 1:**
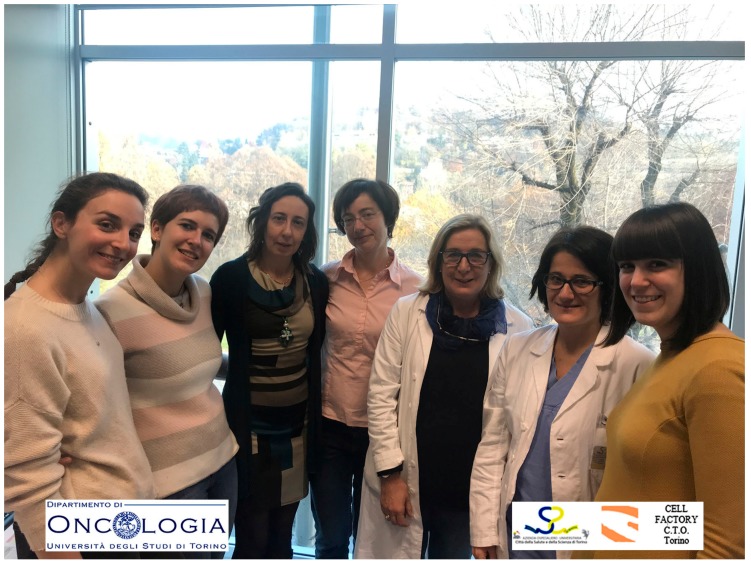
Biochemistry research group, Department of Oncology, in collaboration with Banca della Cute, AOU Città della Salute e della Scienza, University of Torino, Italy. From left to right: Camilla Fiz, Giulia Apprato, Loredana Bergandi, Francesca Silvagno, Carlotta Castagnoli, Daniela Alotto, Chiara Ricca.

**Excessive Endoplasmic Reticulum Stress Correlates with Impaired Mitochondrial Dynamics, Mitophagy and Apoptosis, in Liver and Adipose Tissue, but Not in Muscles in EMS Horses**

Krzysztof Marycz, Katarzyna Kornicka, Jolanta Szlapka-Kosarzewska and Christine Weiss

*Int. J. Mol. Sci.***2018**, *19*(1), 165; doi:10.3390/ijms19010165

Available online: http://www.mdpi.com/1422-0067/19/1/165

Equine metabolic syndrome (EMS) become a cluster of symptoms triggered by obesity, hyperinsulinemia, and hyperleptinemia, increased adiposity, as well as insulin resistance (IR). Although the phenotypic features of horses suffered from EMS are described, the molecular mechanism underlying disease development remains unclear. Thus, in the present study, we used RT-qPCR and western blot methods to compare the expression of genes and proteins involved in apoptosis, IR, and endoplasmic reticulum stress in insulin-sensitive tissues, such as muscles, liver, and adipose tissue. In addition, we estimated mitochondrial dynamics and mitophagy in those tissues, because mitochondrial dysfunction is associated with the development of EMS. Cell ultrastructure was visualized using electron transmission microscopy (TEM). We demonstrated for the first time that liver and adipose tissue isolated from EMS individuals are characterized by enhanced mitochondrial damage and mitophagy, followed by occurrence of apoptosis as mitophagy failure to restore cellular homeostasis. This may indicate that liver and adipose tissue are affected by progressive p53-related apoptosis in EMS and, along with ER stress, promote autophagy to protect the cells against glucolipotoxicity. Nevertheless, in muscles, p53-related apoptosis was decreased, suggesting the existence of a protective mechanism allowing the muscle tissue to maintain homeostasis. Further detailed research is strongly required to explore this issue, since obtained results may be a valuable source of information which may contribute to the development of future clinical therapy of EMS.

**Figure 2:**
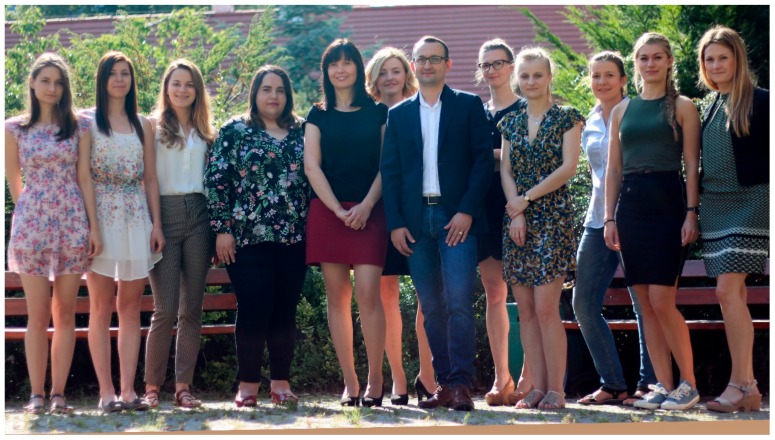
Marycz’s Lab—Reg-Med-Lab Group. Krzysztof Marycz (left seven), Katarzyna Kornicka-Garbowska (left nine) and Jolanta Szłapka-Kosarzewska (left ten).

**CircSMARCA5 Inhibits Migration of Glioblastoma Multiforme Cells by Regulating a Molecular Axis Involving Splicing Factors SRSF1/SRSF3/PTB**

Davide Barbagallo, Angela Caponnetto, Matilde Cirnigliaro, Duilia Brex, Cristina Barbagallo, Floriana D’Angeli, Antonio Morrone, Rosario Caltabiano, Giuseppe Maria Barbagallo, Marco Ragusa, Cinzia Di Pietro, Thomas Birkballe Hansen and Michele Purrello

*Int. J. Mol. Sci.***2018**, *19*(2), 480; doi:10.3390/ijms19020480

Available online: http://www.mdpi.com/1422-0067/19/2/480

Circular RNAs (circRNAs) are a large and functionally heterogeneous group of RNAs recently rediscovered thanks to the combination of RNA sequencing and downstream focused bioinformatic data analysis. Initially thought to be simple byproducts of the splicing process of their host genes’ transcripts, circRNAs are now under the spotlight due the important biomolecular functions they perform within eukaryotic organisms and their cells. Accordingly, it will be crucial to continue to investigate on the molecular mechanisms through which they act to characterize them as: (i) RNAs functionally involved in a disease state; (ii) diagnostic and prognostic biomarkers; (iii) druggable targets. In this scenario, we focused on a circRNA (named circSMARCA5, from the name of its host gene transcript) quite abundantly expressed in the brain in physiological condition. qPCR analysis of biopsies from patients with Glioblastoma multiforme (GBM) and unaffected control tissues revealed the downregulation of circSMARCA5. After cloning its sequence in an expression vector, we also demonstrated its functional involvement in the control of GBM cell migration, by using U87MG cell line as in vitro model. Mechanistically, based on in silico prediction of RNA-protein interactions and publicly available eCLIP (enhanced UV crosslinking and immunoprecipitation) data, we also proposed that circSMARCA5 may exert its function by tethering and sponging the protein *Serine and Arginine Rich Splicing Factor 1* (SRSF1), a splicing factor (SF) known to be involved in promoting tumorigenesis. Aberrant splicing of another SF (*Serine and Arginine Rich Splicing Factor 3*, SRSF3), whose alternative splicing is known to be regulated by SRSFS1, in GBM cell lines transfected with circSMARCA5, added value to our hypothesis. Overall, the data presented in this paper allow to candidate circSMARCA5 as a tumor suppressor circRNA in GBM and propose it as a reliable druggable target due to its role in the regulation of a known tumorigenic SF. 

**Figure 3:**
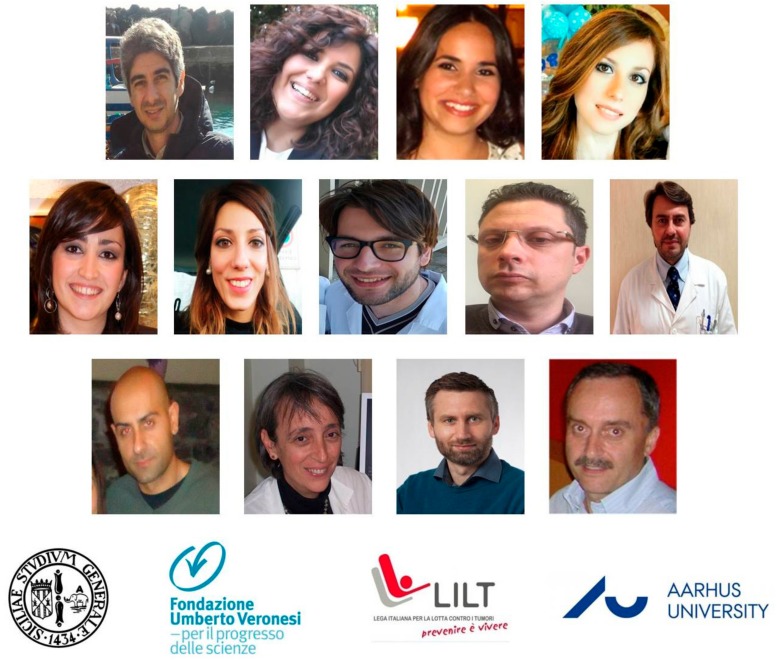
From left to right: Davide Barbagallo, Angela Caponnetto, Matilde Cirnigliaro, Duilia Brex, Cristina Barbagallo, Floriana D’Angeli, Antonio Morrone, Rosario Caltabiano, Giuseppe Maria Barbagallo, Marco Ragusa, Cinzia Di Pietro, Thomas Birkballe Hansen, and Michele Purrello.

## Review Paper Award:

**The Roles of Autophagy in Cancer**

Chul Won Yun, Sang Hun Lee

*Int. J. Mol. Sci.***2018**, *19*(11), 3466; doi:10.3390/ijms19113466

Available online: http://www.mdpi.com/1422-0067/19/11/3466

This paper describes the roles of autophagy in cancer. Our increasing understanding of autophagy has revealed the very complex and sophisticated contribution of autophagy to tumorigenesis. In normal conditions, autophagy is an intracellular degradative process that occurs under several stressful conditions, including organelle damage, presence of abnormal proteins, and nutrient deprivation. The mechanism of autophagy initiates the formation of autophagosomes that capture degraded components and then fuse with lysosomes to recycle these components. Therefore, the aim of this review paper was to reveal the mechanism of autophagy in biological conditions and describe the biological and pathological roles in cancer. We summarized the typical biological mechanism of autophagy, as well as the role of autophagy in cancer, such as in tumor suppression and promotion. We also described that autophagy regulates the properties of cancer stem cells by contributing to the maintenance of stemness, the induction of recurrence, and the development of resistance to anticancer reagents. Furthermore, we discussed the potential clinical applications of autophagy, especially as novel therapeutic targets in cancer treatment (Figure 1).

**Figure 4:**
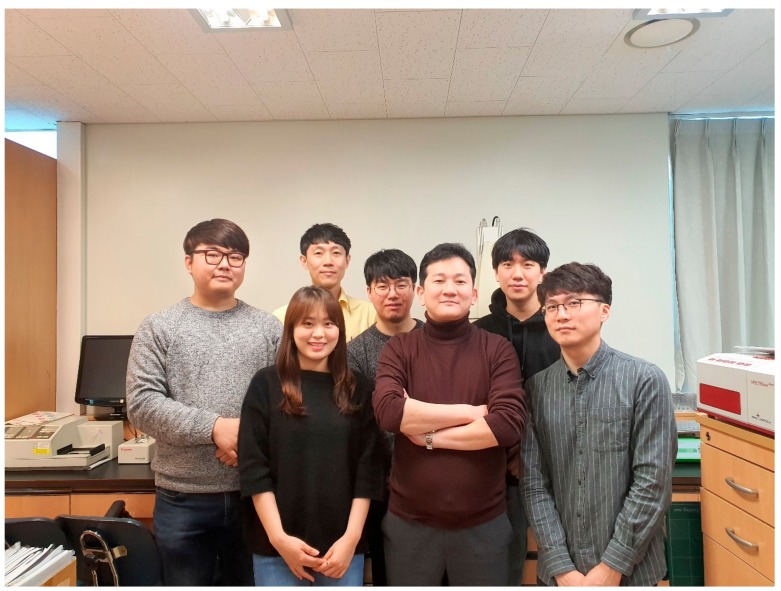
Department of Biochemistry, Soonchunhyang University College of Medicine, Cheonan, Republic of Korea. Chul Won Yun: left one; Sang Hun Lee: left five.

**Noncoding RNA:RNA Regulatory Networks in Cancer**

Jia Jia Chan, Yvonne Tay

*Int. J. Mol. Sci.***2018**, *19*(5), 1310; doi:10.3390/ijms19051310

Available online: http://www.mdpi.com/1422-0067/19/5/1310

Noncoding RNAs constitute a large proportion of the human transcriptome. Despite this, they were previously regarded as “junk” due to their lack of coding potential. Insights into their functions and physiological relevance only began to emerge in the last two decades. This review focuses on the regulatory networks in cancer involving various noncoding RNA species termed “competing endogenous RNA (ceRNA) networks”. This phenomenon describes the competition between coding and noncoding RNAs for binding to specific microRNAs for which they have binding sites. As conventional microRNA binding is known to repress target expression, this competitive binding leads to the bidirectional regulation of expression of these ceRNAs and their downstream functions. Here, we comprehensively summarized the roles of various long noncoding RNAs, pseudogenes and circular RNAs in both oncogenic and tumor suppressive ceRNA axes. We highlighted the importance of stoichiometric balance and dynamic cellular localization that allow the different components to come together as part of the same ceRNA network upon specific physiological and extracellular cues. More importantly, with the recent advances in high throughput sequencing and computational platforms, many research groups are adopting an integrative computational approach to identify ceRNA interactions with clinical relevance, specifically ceRNA axes that could function as robust biomarkers and predict drug responses in a cancer-specific manner. This approach, coupled with cutting edge experimental techniques, including the CRISPR-Cas system, could be harnessed to advance the field of noncoding RNAs and ceRNA regulation to provide more detailed insights into their mechanistic functions and clinical values, facilitating more efficient “bench to bedside” translational research. 

**Figure 5:**
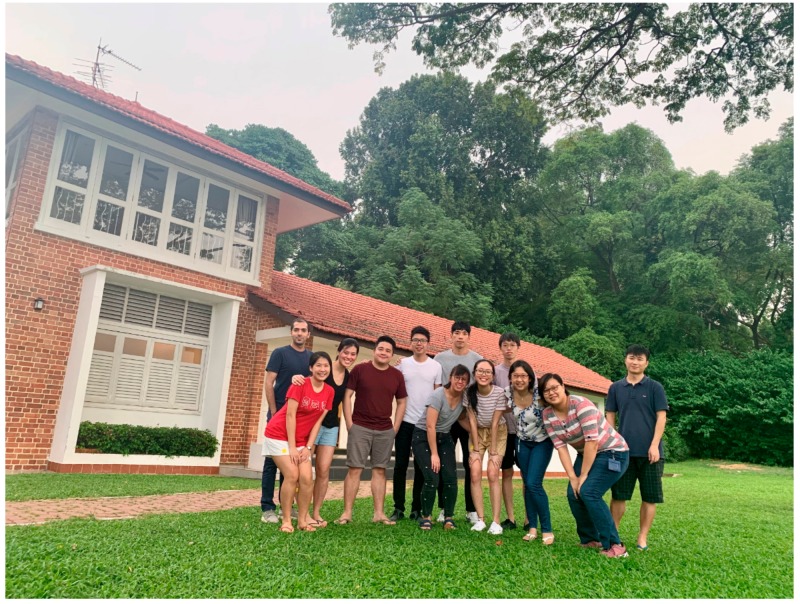
Yvonne Tay lab: Jia Jia Chan (left three), Yvonne Tay (right three).

**The Role of NADPH Oxidases and Oxidative Stress in Neurodegenerative Disorders**

Anuradha Tarafdar, Giordano Pula 

*Int. J. Mol. Sci.***2018**, *19*(12), 3824; doi:10.3390/ijms19123824

Available online: http://www.mdpi.com/1422-0067/19/12/3824

In this review, we discussed the recent developments in our understanding of the mechanisms linking nicotinamide adenine dinucleotide phosphate (NADPH) oxidases (NOX) activity, oxidative stress (OS), and neurodegenerative diseases, with particular focus on the neurovascular component of these conditions. For a long time, NOX enzymes were considered to be a peculiarity of professional phagocytic cells. However, over the last decade, several homologs have been identified and based on current research the NOX family consists of NOX1, NOX2, NOX3, NOX4, NOX5, DUOX1, and DUOX2 enzymes. These enzymes have unique distribution patterns and expression levels in different tissues. NOXs are membrane-bound proteins with the main function of transferring electrons across the plasma membrane to molecular oxygen-which results in the generation of the reactive oxygen species (ROS)-primarily superoxide anion (O_2_^●−^), although hydrogen peroxide (H_2_O_2_) can also be generated. Elevated ROS leads to OS, which has been associated with a myriad of inflammatory and degenerative pathologies. OS is also the commonality in the pathophysiology of neurodegenerative disorders, such as Alzheimer’s disease (AD), Parkinson’s disease (PD), Huntington’s disease (HD), amyotrophic lateral sclerosis (ALS), and multiple sclerosis (MS).

**Figure 6:**
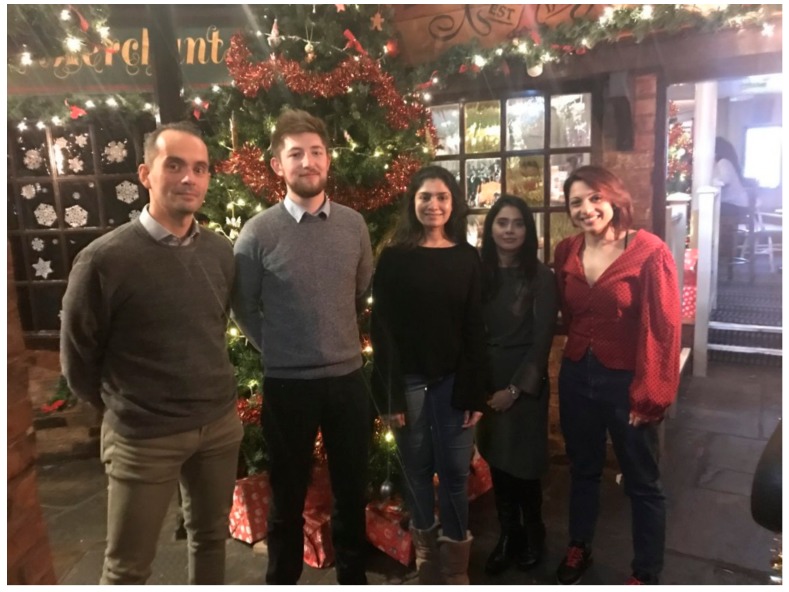
Left to right: Giordano Pula, Stuart Wallis, Aishwarya Vaidya, Dina Vara and Anuradha Tarafdar.

Dr Pula’s lab (Figure 6) is interested in understanding NADPH-redox signaling and OS in the vasculature during physiological and pathophysiological conditions. With this review, we aimed to summarize published evidence linking OS and central nervous system (CNS) degeneration, with particular focus on our understanding of the role of NOX enzymes in neurovascular disorders and the current challenges hampering effective pharmacological targeting of NOXs.

We believe that these six exceptional papers are valuable contributions to the *International Journal of Molecular Science* and the scientific research field. On behalf of the *International Journal of Molecular Science* Editorial Board, we would like to congratulate these teams for their excellent work. In recognition of their accomplishment, they will receive the privilege of publishing an additional research article or review paper free of charge in open access format in the *International Journal of Molecular Science*, after the usual peer-review procedure.

We would like to take this opportunity to thank all the nominated research groups of the aforementioned exceptional papers for their contributions to the *International Journal of Molecular Science*, and thank the *International Journal of Molecular Science* Editorial Board for voting and helping with this “Best Paper Award”.

The Editorial Board and Editorial Staff at the *International Journal of Molecular Science* is committed to meeting the needs of the molecular research community by providing useful and timely reviews of all manuscripts submitted, and providing an open access journal for your results. Please consider submitting your work to the *International Journal of Molecular Science*, and we look forward to announcing your paper as an *International Journal of Molecular Science* Best Paper in the future.

## Prize Awarding Committee

*International Journal of Molecular Science* Editorial Board.
